# Supplementary material for: Health-related quality of life of adults with cutaneous leishmaniasis at ALERT Hospital, Addis Ababa, Ethiopia
Source: PLoS Negl Trop Dis. 2023 Oct 30;17(10):e0011196. doi: 10.1371/journal.pntd.0011196 (PMC10645367; doi:10.1371/journal.pntd.0011196)
Supplement: Attachment — Submitted filename: DLQI response to comments.docx [file pntd.0011196.s001.docx]

To the editorial office

PLOS NTD

Re: PNTD-D-23-00246

Dear Editor,

We are grateful to the reviewers for their time and insightful comments. We have replied to all their comments section-by-section and reviewer by reviewer.

Sincerely

Shimelis Doni, on behave of the investigators’ team

**Methods**

**Reviewer #1**:

**Comment**: This paper is clearly written, and the way the study is described should make it replicable. The population chosen is large enough to come to useful conclusions, and to pinpoint differences based on gender (which turn out to be important in the conclusions).

***Response:*** *Thank you,*

**Reviewer #2**:

**Comment**: In the lines 104-106, the authors present all DLQI domain. However, in the results (Table 4) the treatment domain was not considered. This point must be detailed.

**Response:** *Thank you so much for the comment. All domains are included in our description of the DLQI including the treatment domain (Question 10) please see Line 108 and 109 “……and treatment (Question 10).” In addition, in table 4 all six DLQI domains are presented, Page 9, lines 167-178.*

**Comment**: Which moment the patients answered the questionnaire (before, during or after the treatment)? This is an important point that could affect the HRQoL of the patients, especially considering the CL-treatment limitations. This information could be better described in the methodology section.

**Response:** *Thank you. We agree and have clarified the timing of the completion of the DLQI with respect to treatment. Line 104-105 now reads…… “The validated Amharic version of the DLQI [17] was completed for each participant prior to start of treatment by a trained interviewer.”*

**Reviewer #3:**

**Comment**: the hypothesis and objectives are not clear.

**Response:** *The objective is stated on Lines 90-91. We have edited the sentence to read “We wished to assess the HRQoL associated with CL in a referral hospital setting in Ethiopia using the DLQI.”*

**Comment**: Was the inclusion consecutively? No information about it.

**Response:** *No. Individuals were invited to participate when staff were available to take consent and administer the DLQI.*

**Comment**: Not all the variables are defined and explained example: sex and residence.

**Response:** *We believe this is clear***.**

**Comment**: How did they calculate the sample size?

**Response:** *No formal sample size calculation was performed and we wished to recruit as many individuals as possible in the time period available. Our cohort is larger than other comparable studies.*

**Comment**: *Explain which groupings are chosen for comparisons and why example: between leishmaniasis types.*

**Response:** *Line 99-101 ….“* The treating health care professionals classified participants as having LCL, MCL or DCL. Demographic and clinical data including location of lesions were recorded on a data collection form.*”*

**Comment**: How were missing data addressed?

**Response:** *There was no missing data as the DLQI was administered by an interviewer.*

**Comment**: How much data was missing per variable?

**Response:** *There was no missing data as the DLQI was administered by an interviewer.*

**Comment**: Number of Patients eligible, included, reasons for exclusion: Consider a flow diagram.

**Response:** *We did not keep data on individuals who did not wish to participate which was the only exclusion criteria. We do not feel a flow diagram would make our manuscript clearer.*

**Comment**: The multivariate model contains related variables (DCL and multiple body parts) and the arguments to enter variables are not mentioned in the methods.

**Response:** *DCL and multiple body regions are not necessarily related. Diffuse CL can be located on a single body region.*

**Comment**: The last references are not well formatted.

**Response:** *We apologize for this and have addressed this.*

**Results**

**Reviewer #1:**

**Comment**: Results are clearly presented; the figures are helpful in visualising these differences.

**Response:** *Thank you.*

**Reviewer #2:**

**Comment**: *I suggest to the authors to show the* ***median score of each DLQI domains according*** *the clinical phenotype; was any domain more affected by clinical forms?*

**Response**: thank you for this, we have included table 5, “. Percentage of total DLQI domain score and median/ Interquartile range by clinical phenotype of participants.” See page 9, lines 171-173

**Reviewer #3:**

**Comment**: Please explain what is COR and what is AOR in table 5

**Response**: *Thank you. We have included a footer to the table as “^*^COR= crude odds ratio, ^**^AOR= adjusted odds ratio”; please see line 184*

**Conclusions**

Reviewer #1:

**Comment**: The conclusions seem to flow very well from the findings, and are compared with literature in a useful way.

**Response**: *Thank you.*

**Reviewer #2:**

**Comment**: I think that the conclusion presented should be more cautious since the HRQoL assessment was not carried out according to the treatment, or after the treatment.

**Response*:*** *Thank you. We agree and have extensively edited the discussion to emphases that our study illustrates associations and that further work is required to elucidate the full impact of the condition.*

**Reviewer #3:**

Comment: The authors do not present a comparison with healthy, non-CL, or cured patients. The authors have no background score for healthy individuals and therefore cannot conclude that the `The HRQoL impairment in people affected by CL is significant´ we have to remember that such scores are highly culturally dependent.

**Response**: *Thank you for this comment. Given the published so far, DLQI score use and interpretations to date “*[*https://www.cardiff.ac.uk/medicine/resources/quality-of-life-questionnaires/dermatology-life-quality-index#:~:text=The%20DLQI%20is%20calculated%20by,affected%20by%20their%20skin%20disease*](https://www.cardiff.ac.uk/medicine/resources/quality-of-life-questionnaires/dermatology-life-quality-index#:~:text=The%20DLQI%20is%20calculated%20by,affected%20by%20their%20skin%20disease)*.” We believe that we made appropriate use of the tool and it is reasonable to claim that CL is associate with significant HRQoL impairment.*

**Editorial and Data Presentation Modifications?**

**Reviewer #1:** There are some occasional errors in English usage, so the paper could benefit from a careful proofreading run (for example: see lines 199-202).

**Response**: Thank you. We have carefully read the manuscript and attended to the language;

Reviewer #2: NA

**Reviewer** #3: Line 57 and 58 improved, counseling on the nature of CL, therapeutic options as well as clinical outcomes and complications. This sentence is not correct.

**Comment**: Table 1 Clinical fenotype DCL percentage contains an error.

**Response**: Thank you so much a comment “we have not used the word …..*fenotype*…… in no part of our manuscript, rather we used *Clinical phenotype to mean clinical presentation*. Also, we could not get the message “improved, counseling on the nature of CL, therapeutic options as well as clinical outcomes and complications.” Is not correct.

**Summary and General Comments**

**Reviewer #1**:

**Comment**: This is a useful study, and provides good evidence for use of the DLQI in assessing the life impact of CT and related conditions.

**Response**: **Thank you**

**Reviewer #2:** (No Response)

**Reviewer #3:**

**Comment**: The manuscript is well written and presented and the topic very well chosen. The lack of comparison with healthy patients impairs the interpretation of the results. It would be essential to include such a control group in order to conclude how CL affects people. The article might focus on the differences between groups such as: male/female, urban/rural, etc. As it is now, the article doesn’t provide novel information that helps to improve our understanding of CL related HRQL. Finally, qualitative interviews would enrich the interpretation of the findings.

**Response**: *Thank you! We have focused our interpretation on the data. On how to use/who can use DLQI is present* [*https://www.cardiff.ac.uk/medicine/resources/quality-of-life-questionnaires*](https://www.cardiff.ac.uk/medicine/resources/quality-of-life-questionnaires)*. Our data are novel as HRQoL in CL hardly exits in Ethiopia before. We agree that further work could enrich our understanding of CL in Ethiopia and we are doing this and recommend others too. Moreover, we believe our data are important for researchers and policy makers to have an insight into the quality of life associated with CL.*
