# Supplementary material for: Health-related quality of life of adults with cutaneous leishmaniasis at ALERT Hospital, Addis Ababa, Ethiopia
Source: PLoS Negl Trop Dis. 2023 Oct 30;17(10):e0011196. doi: 10.1371/journal.pntd.0011196 (PMC10645367; doi:10.1371/journal.pntd.0011196)
Supplement: Attachment — Submitted filename: Doni et al response to reviewers comment.docx [file pntd.0011196.s002.docx]

To the editorial office

PLOS NTD

Re: PNTD-D-23-00246

9^th^ August 2023

Dear Editor,

We are grateful for the opportunity to submit the revised manuscript “Health-Related Quality of Life of Adults with Cutaneous Leishmaniasis at ALERT Hospital, Addis Ababa, Ethiopia”.

We appreciate the reviewers’ comments on our manuscript. We have addressed these comments within the revised manuscript.

Please see below, our point-by-point response to the reviewer’s comments and concerns.

Sincerely

Shimelis Doni, on behalf of the authors

Comment and Response

**Reviewer #3:**

**Comment 1:** the hypothesis and objectives are not clear.

**Response 1**:

*On line 87 we state:*

*“We aimed to assess the HRQoL associated with active CL” and in the methods we detail how we did this.*

**Comment 2**: Was the inclusion consecutively? No information about it.

**Response 2:**

*Thank you we have clarified this point. We now state on Line 95 that “Inclusion was not consecutive as staff were not always available to recruit and not all affected individuals spoke Amharic.”*

**Comment 3**: Not all the variables are defined and explained example: sex and residence.

**Response 3:**

*We have further clarified these variables on Line 118:* “*sex (male or female), age (in years), and residence (urban or rural identified according to Kebele of residence)”*

**Comment 4:** How did they calculate the sample size?

## Response 4:

## *No formal sample size was calculated.*

**Comment 5:** Explain which groupings are chosen for comparisons and why example: between leishmaniasis types.

**Response 5:**

*The different clinical phenotypes of cutaneous leishmaniasis present in Ethiopia are long established and so these were chosen to determine if these individuals had different health-related quality of life as measured by the DLQI. Other studies have examined different types of skin lesions and so using clinical phenotype in this type of study is not unreasonable.*

**Comment 6:** How were missing data addressed? How much data was missing per variable?

**Response 6:**

*We have included the statement Line 120 “Participants with missing data were to be excluded but no data were missing.”*

**Comment 7:** Number of Patients eligible, included, reasons for exclusion: Consider a flow diagram.

**Response 7:**

*Only data on individual who participated in the study was kept. We do not believe a flow chart will improve the reader’s ability to interpret our data further. We believe we meet the requirements of STROBE sufficiently.*

**Comment 8:** The multivariate model contains related variables (DCL and multiple body parts) and the arguments to enter variables are not mentioned in the methods.

**Response 8:**

*DCL and multiple body regions are not necessarily related. Diffuse CL can be located on a single body region.* LCL and DCL, both do not involve the mucosa.

**Comment 9**:

The authors do not present a comparison with healthy, non-CL, or cured patients. The authors have no background score for healthy individuals and therefore cannot conclude that the `The HRQoL impairment in people affected by CL is significant´ we have to remember that such scores are highly culturally dependent.

**Response 9**:

We used the validated Amharic version of the DLQI which is cited in manuscript*. The DLQI is not a generic measure of HRQoL but specific to individuals with skin disease and as such individuals with no skin disease would not score on questions which assess the impact of skin disease and treatments for skin disease. We disagree that high DLQI scores are not significant and the use of the validated Amharic version is culturally appropriate for our setting.*

**Reviewer #2:**

Comment 1: Dear Editor, I would like to express my appreciation for submitting the article entitled "Health-Related Quality of Life of Adults with Cutaneous Leishmaniasis at ALERT Hospital, Addis Ababa, Ethiopia" and for allowing me to review it. After careful analysis of the manuscript and considering the responses provided by the authors, I am convinced that the work is well-structured, presents a significant study, and should be published in PLOS Neglected Tropical Diseases.

All of my comments were adequately addressed by the authors, and they made the necessary adjustments to the manuscript based on my suggestions. However, I would like to highlight an aspect that I believe could be further explored and elaborated upon in the discussion section: the choice of the tool used to assess the patients' quality of life.

Assessing the quality of life is a crucial component in studies aiming to understand the impact of diseases on patients' lives. I am pleased to see that the authors selected a specific tool for this purpose in their study. However, I believe it would be valuable if the authors dedicated more discussion space to describing the selection of the tool and discussing its advantages. Additionally, they could explore how this tool compares to other available options.

Once again, I would like to commend the authors for their work and thank you for the opportunity to review this article. I hope that my suggestions will be helpful in further improving the manuscript.

**Response** 1:

*Thank you. We chose a validated Amharic version of the DLQI. We believe a discussion on the use of other tools is beyond the scope of our paper as we did not test other tools. We have indicated that we believe the use of patient reported outcome measures are important in assessing efficacy of treatments and we are currently undertaking this work. We hope to discuss the merits of different tools in future publications.*
